# HPV vaccination is fundamental for reducing or erradicate penile cancer | *Opinion: YES*

**DOI:** 10.1590/S1677-5538.IBJU.2018.05.02

**Published:** 2018

**Authors:** Brunno Raphael Iamashita Voris, Carolina Del Negro Visintin, Leonardo O. Reis

**Affiliations:** 1 Departamento de Urologia, Universidade Estadual de Campinas, Unicamp, Campinas, SP, Brasil; 2 Departamento de Ginecologia, Pontifícia Universidade Católica de Campinas, PUC-Campinas, SP, Brasil; 3 UroScience, Campinas, SP, Brasil; 4 Departamento de Urologia, Pontifícia Universidade Católica de Campinas, PUC - Campinas, SP, Brasil

**Keywords:** Human papillomavirus 31, Penile Neoplasms, Vaccination, Male

Penile cancer is a rare tumor in developed countries; since its diagnosis is made usually in a late stage, it presents high morbidity and mortality. It is more prevalent in developing countries in Asia, Africa and South America, particularly in non-circumcised males (1).

More common histology type is penile squamous cell carcinoma - PSCC (95%) related to the infection by the Human Papillomavirus (HPV) (2). HPV-DNA is found in more than 20% of patients with penile tumor and in 90% of anal cancers (3). The number of new patients with PSCC is increasing worldwide, including developed European countries (Denmark, Netherlands, England), and has been worried many health care professionals around the World (4).

Treatment of PSCC is mainly surgical, with local resection of lesion, partial or total penectomy and occasionally inguinal and eventually pelvic lymphadenectomy. Dissemination is mainly by lymph nodes (inguinal, pelvic) and by bloodstream.

PSCC etiology is multifactorial, including phimosis, poor hygiene, smoking, chronic inflammation (*balanitis xerotica obliterans).* Other important risk factors include: high number of sexual partners, genital wart disease of sexually transmitted diseases (STD) (5).

HPV infection is the most common sexually transmitted disease in the World, and 50% to 80% of sexually active persons have already been infected by that virus at any moment in their life (6). It was the first identified virus related to malign transformation of cells at the uterine cervix at the 70's and, ever since, some serotypes have been correlated to superficial epithelial tumors at the vulva, vagina, anus, penis and oropharynx.

Cervical uterine cancer is also a disease caused by the persistent oncogenic HPV infection. At the uterine canal, the transformation zone, characterized by areas of immature metaplastic squamous cells, is particularly vulnerable to HPV infection. Apparently, virions infect only human cells, in special stratified squamous epithelium. Usually, HPV infection occurs in microcracks at the surface of epithelium, invading basal membrane cells, where the viral genome is introduced in the cell nucleus (7).

HPV infection leads to displasic transformation of basal cells, that may heal spontaneously or evolve to neoplastic precursor cells and developing high grade lesions, resulting in tumors. During carcinogenesis, infection may alter oncotic cytology, also known as uterine cervix Papanicolau exam, that is used as screening method in women. Persistent high risk HPV infection is the key to lesion progression.

HPVs are members of the *Papillomaviridae* family, small non-enveloped virus with double chain of DNA. More than 200 types of HPV have already been identified and 140 have been submitted to DNA sequencing (6). Thirteen are carcinogenic: with high risk to develop displasia and neoplasia (8). HPB subtypes 16 and 18 are responsible for 70% of all malign uterine cervix tumors (4, 9). Serotypes 6 and 11 are less associated to vaginal and penile oncogenesis, but are more associated to vulva, anus and oropharynx carcinogenesis (10). They are also responsible for condylomatosis.

Prevalence of HPV infection in women is around 12% and in men 20% (1.3% – 72.9%). Most patients are uncircumcised. HPV infection is usually asymptomatic and may cause two types of lesions: acuminate condyloma (ano-genital warts), low grade lesions, and tumors, high grade lesions.

Infected patients by serotypes 16 and 18 present high levels of specific antibodies, reducing the risk of subsequent infections by the same serotype. This evidence allowed the development of vaccines with viral parts of each subtype.

The first HPV vaccine approved by the American Federal Drug Administration (FDA) was marketed in 2006. At present, there are three different vaccines: bivalent (2014), that targeted the infection of serotypes 16 and 18; quadrivalent (targeting serotypes 6, 11, 16 and 18); and nonavalent (last version), that protects against infection of subtypes 6, 11, 16, 18, 31, 33, 45, 52 and 58 (6, 11).

Dosage of antibodies in vaccinated patients with bivalent or quadrivalent vaccines, compared to patients infected by HPV is higher. In that group of vaccinated patients, 93% to 100% showed seroconversion. It was showed also that monovalent vaccine, when administered in two doses, has the same efficacy than when three-times administered. In a random trial, patients with high risk lesions that were vaccinated, compared to a control group, showed less risk of malign transformation. Also, it was demonstrated that vaccine efficacy is higher when administered before the beginning of sexual life of patients (6).

In patients with more that 15 years old, the vaccination program is performed in three stages: initial dose, followed by a second dose after 1-2 months and final dose after 6 months. Before 15 years old or the beginning of sexual life, vaccination must be administered in two doses, with interval of 6-12 months (12). Men with more than 26 years of life that have sex with other men or that are immunosuppressed must also be vaccinated (13).

The proposal and dissemination of HPV vaccine and studies that showed correlation of HPV infection and several tumors have increased the use of vaccine in several countries (2). For example, in the US, recent data show that in 2015, 28% of men completed the three-dose vaccine plan and also 41.9% of women and 34.9% of adolescents (14).

HPV vaccination showed good results in women, and is safe and efficient to prevent uterine cervical cancer. In men, vaccine efficacy was high in studies that included non- -treated men against HPV (15).

World Health Organization strongly recommends vaccination of children with both sexes. After the beginning of vaccination, rate of infection reduced. In the US, it is recommended vaccination of children, and men and women from 9 to 26 years old (16). It is also recommended screening of cervical uterine cancer using Papanicolau exam to evaluate oncotic cytology.

HPV vaccination became an excellent tool to prevent virus infection, and therefore, prevention of neoplastic diseases in men (preventing penile cancer and also transmission of virus to women, consequently preventing uterine cervix, ano-genital and oropharynx tumors related to HPV infection).

The number of women, adolescents and young adults vaccinated against HPV is increasing worldwide, due to the increase of patients infected by HPV (including developed countries), the correlation of HPV and tumor in both sexes showed in recent studies, and the availability and easy of access to vaccination of young adults and adolescents, with direct impact in the prevention of neoplastic diseases of oropharynx, anus and genitals, such as uterine cervix and penis.

Therefore, it is recommended vaccination of adolescents (boys and girls) with 11-12 years old, or before the beginning of sexual life (preferably) to prevent direct infection of HPV in both sexes, and to prevent cross-infection, actively preventing neoplastic diseases beyond penile tumors, such as ano-genital (vulva, uterine cervix and anus) and oropharynx cancers.
